# A new vector-based global river network dataset accounting for variable drainage density

**DOI:** 10.1038/s41597-021-00819-9

**Published:** 2021-01-26

**Authors:** Peirong Lin, Ming Pan, Eric F. Wood, Dai Yamazaki, George H. Allen

**Affiliations:** 1 Department of Civil and Environmental Engineering, Princeton University, Princeton, NJ 08544 USA; 2 Institute of Industrial Science, University of Tokyo, Tokyo, Japan; 3 Department of Geography, Texas A&M University, College Station, Texas USA

**Keywords:** Hydrology, Environmental sciences

## Abstract

Spatial variability of river network drainage density (*D*_*d*_) is a key feature of river systems, yet few existing global hydrography datasets have properly accounted for it. Here, we present a new vector-based global hydrography that reasonably estimates the spatial variability of *D*_*d*_ worldwide. It is built by delineating channels from the latest 90-m Multi-Error-Removed Improved Terrain (MERIT) digital elevation model and flow direction/accumulation. A machine learning approach is developed to estimate *D*_*d*_ based on the global watershed-level climatic, topographic, hydrologic, and geologic conditions, where relationships between hydroclimate factors and *D*_*d*_ are trained using the high-quality National Hydrography Dataset Plus (NHDPlusV2) data. By benchmarking our dataset against HydroSHEDS and several regional hydrography datasets, we show the new river flowlines are in much better agreement with Landsat-derived centerlines, and improved *D*_*d*_ patterns of river networks (totaling ~75 million kilometers in length) are obtained. Basins and estimates of intermittent stream fraction are also delineated to support water resources management. This new dataset (MERIT Hydro–Vector) should enable full global modeling of river system processes at fine spatial resolutions.

## Abstract (summary)

**Table:** 

Measurement(s)	vector river network • river network drainage density
Technology Type(s)	computational modeling technique • machine learning
Sample Characteristic - Environment	river • watershed • drainage basin
Sample Characteristic - Location	global

Machine-accessible metadata file describing the reported data: 10.6084/m9.figshare.13377329

## Background & Summary

High-accuracy hydrography data delineating global river networks and basin boundaries lay the foundation for many important geoscience applications, such as global hydrologic modeling^1–3^, ecohydrological analysis^4^, geomorphological analysis^5^, and water resources management^6^. During the past two decades, improvements in the resolution and accuracy in spaceborne digital elevation models (DEMs) have greatly advanced the delineation of such hydrographic data – prominent recent examples include the HydroSHEDS^7^ benchmarking the global hydrography dataset since the release of the Shuttle Radar Topography Mission (SRTM)^7^, and its recent variant HydroATLAS^2^ that contains millions of river flowlines with hydro-environmental information.

Despite these promising developments, a drawback common to existing global hydrography datasets is a lack of proper consideration of channelization thresholds that vary across different climatic and physiographic conditions. Determining the controls of channelization threshold is a fundamental topic in the field of geomorphology which has been widely studied^8–10^. Yet as of today, it remains an open scientific challenge^11,12^, and despite various strategies for small-scale hydrography delineations based on the area-slope relationships^9^ or physical constraints^5^, there is still a lack of methods and consistent reference data that can lead to a satisfactory solution at the global scale. As a result, existing global hydrography datasets often do not present river network drainage density (*D*_*d*_) reasonably. *D*_*d*_ is defined as the unit length of channel networks within a specific area [L^−1^]. It describes the drainage network texture, which determines the flow concentration time by defining the length of the stream network and hillslope paths^13^. Subsequently, *D*_*d*_ can influence the accuracy of hydrologic modeling especially flood modeling.

For example, the most widely-used, publicly-available global hydrography dataset, HydroSHEDS, used a constant flow accumulation area threshold of 100 pixels to delineate channel flowlines^7^. Lin *et al*.^3^ adopted a similar method to delineate ~3 million river reaches globally and constructed a global river routing model, where *D*_*d*_ of the river network does not vary across regions. Recently, HydroSHEDS was updated to adopt a finer threshold (0.1 m^3^/s or 10 km^2^) to map global free-flowing rivers^14^. The updated river network has a total length of 35.9 million kilometers, which represents, to our knowledge, the state-of-the-art vector-based global hydrography today. However, it is important to note that these threshold values were highly empirical, and no evidence was presented as to whether reasonable *D*_*d*_ can be achieved. In addition, HydroSHEDS was based upon the SRTM DEM, which suffers from not covering 60°N and above (thus lacking reliable river network delineation in Arctic basins^15^), and exhibiting multiple error terms related to biases in the topographic data retrieval^16^. These have limited the usefulness of HydroSHEDS in supporting fine-scale geosciences applications such as hyper-resolution hydrologic modeling that emphasizes small streams^17,18^.

To address these limitations, this study develops a new vector-based global hydrography dataset using the latest DEM data and a machine learning method to estimate spatial variability of *D*_*d*_ globally. A high-resolution high-accuracy DEM (3 s, ~90 m) that removes multiple error components, named the Multi-Error-Removed Improved-Terrain (MERIT) DEM^16^, is jointly used with the raster flow direction/accumulation field in MERIT Hydro^19^ as the underlying data layers for global river network extraction. Our machine learning method is based upon geospatial analyses that survey the watershed-scale climate and physiography conditions globally to estimate *D*_*d*_. The newly developed global hydrography is a vector version of Yamazaki *et al*.^19^ and an update to Lin *et al*.^3^, which now considers spatial variability of *D*_*d*_, with ~58 million river flowlines (totaling ~75 million kilometers of rivers globally), 156,571 watersheds, and 57,025 basins (Table 1) to support water resources management.

**Table 1:** Data products including basins, watersheds, river network with variable *D*_*d*_ *(estimated with machine learning)*, and river network with constant *D*_*d*_ (25 km^2^ threshold).

Data	Attributes	Type	Objects/File Size
Basins	*basid*: basin ID *areasqkm*: basin area in km^2^	Polygon shapefile	57,025 basins
Watersheds	*basid*: watershed ID *areasqkm*: watershed area in km^2^ *Q*_*MEAN*_: mean runoff (mm d^−1^) *AI*: aridity index *SND*: sand mass percentage (%) *CLY*: clay mass percentage (%) *SLT*: silt mass percentage (%) *WTD*: water table depth (m below surface) *LAI*: leaf area index *topo*: mean elevation (m) *topo*_*std*_: standard deviation of elevation (m) *urban*: urban fraction (%) *K*: bedrock hydraulic conductivity (m s^−1^) *P*: bedrock porosity (%) *D*_*d*_: estimated drainage density	Polygon geodatabase	156,571 watersheds
River network (variable *D*_*d*_)	*LINKNO*: river ID *strmOrder*: Strahler stream order *strmDrop*: drop in stream (m) *lengthkm*: river length in km *slope*: river slope (km km^−1^) *PFAF_ID*: first two codes of Pfafstetter ID fromnode: integer for the starting point of the river segment tonode: integer for the ending point of the river segment	60 polyline shapefiles (excluding Greenland)	~58 million river flowlines
River network (constant *D*_*d*_, 25 km^2^ threshold)	*LINKNO*: river ID *strmOrder*: Strahler stream order *strmDrop*: drop in stream (m) *lengthkm*: river length in km *slope*: river slope (km km^−1^) fromnode: integer for the starting point of the river segment tonode: integer for the ending point of the river segment	61 polyline shapefiles	~2.9 million river flowlines

*LINKNOs are separately defined in the variable D_d_ and constant D_d_ river network datasets.

Our dataset is validated against Landsat-derived river centerlines, more specifically the Global River Width from Landsat (GRWL) database^20^, at ~50 million locations to demonstrate its improved centerline accuracy. In addition, high-quality regional hydrographic geofabrics are used to validate the estimated *D*_*d*_ patterns, including the United States NHDPlusV2^21^, the Australia Hydrological Geospatial Fabric^22^, and several field-informed geospatial river network data. While rivers can expand/shrink during wet/dry conditions^23^, we note that determining the dynamically-varying channel heads is beyond the scope of this study. The actual locations of channel heads that can be surveyed from field studies^11^ is also beyond our target, because the spatial resolution of the best global DEM data intrinsically constrains us from doing so. Thus, our approach balances the consideration of densified global river network with acceptable computational costs while attempting to approach the maximum DEM-resolvable headwaters.

## Methods

**Fig. 1:**
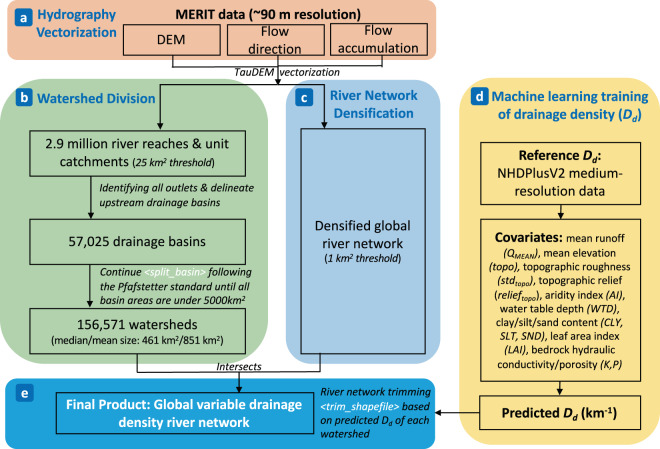
Technical workflow for deriving a high-resolution, high-accuracy global hydrography dataset with variable drainage density. *D*_*d*_ denotes drainage density in the unit of *km*^−1^. The MERIT DEM and MERIT Hydro are obtained from Yamazaki *et al*.^16^ and Yamazaki *et al*.^19^. NHDPlusV2 data are from https://nhdplus.com/NHDPlus/. TauDEM software are from https://github.com/dtarb/TauDEM. < >encloses the Python functions developed for efficient global processing, which are shared at https://github.com/peironglinlin/Variable_drainage_density.

The workflow of our methodology and data generation process is summarized in Fig. 1. In the following sections, the data and methods to vectorize river flowlines and unit catchments, divide watersheds, and estimate variable drainage density are described in detail.

### Hydrography vectorization and underlying data sources

Fig. 1a summarizes the steps of hydrography vectorization. The underlying DEM data we use is the MERIT DEM at 3 arcsec resolution (~90 m), which has demonstrated much improved accuracy over the SRTM DEM^16^ after removing multiple error components such as the absolute bias, stripe/speckle noise, and tree height biases. It combined the Advanced Land Observing Satellite World 3D-DEM (AW3D DEM) to fill in the SRTM gap, providing a much better data source for the Arctic region compared to Hydro1K as currently adopted by Grill *et al*.^14^. Yamazaki *et al*.^19^ recently used the MERIT DEM and several other map layers to compute the MERIT Hydro, a product including the raster flow direction and flow accumulation fields. We use both MERIT DEM and MERIT Hydro as the underlying data sources for our ensuing river network extraction/vectorization as they represent the latest development in global DEM analyses with well documented accuracy assessments^16,19^.

The vectorization of river flowlines and catchments involves the following geospatial analyses: (1) specifying a threshold value to define stream cells, which are grid cells with a flow accumulation exceeding a pre-defined threshold, (2) determining catchment cells based on the location of stream cells and the flow direction field, and (3) extracting the coordinate information for stream cells and catchment cells, which can be used to convert the cells into stream polylines and catchment polgyons. While one can use the widely used ArcHydro tool or any programming language to accomplish these tasks, to deal with ~90 m data globally, we choose to use the TauDEM software’s (http://hydrology.usu.edu/taudem/taudem5/index.html) “*StreamNet*” function (https://hydrology.usu.edu/taudem/taudem5/help53/StreamReachAndWatershed.html) because of its well supported parallel functionality compatible with high-performance computing clusters, which can deal with the huge computation (i.e., requiring hundreds of gigabytes of computer memory) efficiently.

### Methods for watershed division

Fig. 1b shows our watershed division method. To perform hydrography vectorization, we use the level-02 global basin definitions by HydroBASINS (https://hydrosheds.org/images/inpages/HydroBASINS_TechDoc_v1c.pdf) to roughly re-organize the data into 61 global river basins, because these are more hydrologically meaningful units for hydrography extraction, compared to the original data organized as 1150 5° × 5° tiles. In addition, organizing data into 61 basins allows for efficient computation as allowed by the computer memory. We note, however, that the HydroBASINS basin boundaries (sourced from ~500 m SRTM data) are different from those defined by the ~90 m MERIT data. Thus, the new basins need to be re-defined. After the river networks and catchments are first extracted within the rough boundaries, all the most downstream river segments (or outlet points) within each rough basin boundary are identified (globally there are 57,025 of such outlet points). They are then traced back upstream to determine the upstream drainage boundaries. These drainage polygons are dissolved (i.e., combined) if their outlets are within the same level-02 HydroBASINS boundary, which eventually re-defines the 61 global basins.

The 57,025 drainage basins upstream of the global outlet points are further split into smaller watershed units, upon which the variable *D*_*d*_ is applied globally. To divide the watersheds, we follow the Pfafstetter coding^24^ as it is the most widely used methodology for coding and referencing nested hierarchical global river basins. The Pfafstetter coding uses nine-digit algebra to indicate the topological information of the river network and their locations, e.g., even for tributaries and odd for main stems; the larger the number is, the farther away it is from the basin outlets. For level-01 to level-03 Pfafstetter coding that requires grouping of continental basins where subjective decisions are needed to determine the complex continent break-out, we follow the definition of HydroBASINS^25^ to assign the codes. Starting from level-03, the Pfafstetter codes are assigned following Verdin & Verdin^24^. The stopping criteria for the hierarchical watershed splitting is imposed until all basin areas are under 5000 km^2^, because imposing this criterion would eventually lead to 156,571 watersheds with a median size of 461 km^2^ (Fig. S1), which is considered as the reasonable size to apply variable *D*_*d*_ following some pre-assessments explained in Section 2.4, Text S1, & Fig. S2. This level of watershed is approximately equivalent to HydroBASINS^25^ level-08 classification (median size: 475.7 km^2^).

### River network densification

Before variable *D*_*d*_ is applied, we first top the best resolvable *D*_*d*_ by delineating a densified river network globally with a consistent 1 km^2^ channelization threshold, also referred as the river network densification step (Fig. 1c). The threshold is chosen because 1 km^2^ approximates to ~100 pixels, below which the delineated channel networks are believed to have large uncertainties while huge computations are also involved. Thus, we do not go below this threshold noting that it is already finer than existing global studies;^2,3,14,15^ some geomorphology and ecohydrology applications may require even finer river network depictions^11,19^ but they are beyond our scope. The generated dense river networks are separated by the 156,571 watersheds, and then the river network within each watershed is trimmed such that it has a *D*_*d*_ of that estimated by machine learning (ML)_._

### Machine learning estimation of D_d_

To estimate watershed-by-watershed *D*_*d*_ with ML (Fig. 1d), we first select a high-quality regional hydrographic framework for training and referencing. The US National Hydrography Dataset Plus version 2 (NHDPlusV2) 1:100,000 data is chosen, because it has gone through decades of development efforts by the US Geological Survey (USGS) and the US Environmental Protection Agency (EPA)^21^ where extensive ground-truthing was involved^26^. NHDPlusV2 also served as the underlying geofabric for many important applications including the US National Water Model^27–29^. Although we notice the NHDPlusV2 channel headwater areas show some patchy patterns (Fig. S2a & Text S1), these are recognized as inevitable because almost all regional hydrography datasets will involve subjective decisions on “where channel starts”^26,30^. Therefore, NHDPlusV2 is selected for its reasonable spatial patterns of *D*_*d*_^31^ as well as its large spatial extent covering a wide range of climatic and physiographic conditions (Figs. S3, S4). We choose the Hydrologic Unit Region level 10 (HUC-10) classification as the basic unit to train *D*_*d*_, because it, with a median size of 470.21 km^2^, leverages the consideration of the watershed size representativeness as well as the computational constraints (Text S1 & Fig. S2b). This has also led to our decision of splitting the global basins into a few hundred square kilometers in size, similar to HUC-10, to apply the variable *D*_*d*._ Fig. S3 shows the spatial patterns of the median headwater drainage area, *D*_*d*_ accounting for both perennial and intermittent streams, the perennial *D*_*d*_, and the fraction of intermittent streams (*f*_*i*_) at HUC-10 level.

We select several covariates to estimate the spatial variability of *D*_*d*_ based on our physical knowledge on what potentially controls *D*_*d*_. This includes a range of climatic, topographic, hydrologic, and geologic factors; Text S2 and Figs. S4, S6 will introduce more details of these factors, their spatial patterns, and the interpretations on their relationships with *D*_*d*_. We use a boosted gradient tree-based regressor XGBoost^32,33^ to train and optimize the ML model with five-fold cross validation. After obtaining a reasonably good prediction for the training/validation data (Fig. S7), the optimized ML model is used to estimate *D*_*d*_ globally.

### River network trimming and generation of final data product

To generate the final variable *D*_*d*_ hydrography data product, the last step is to trim the dense river network produced in *Section 2.3* based on ML-estimated *D*_*d*_ (the trimming process is summarized in Fig. 1e). More specifically, for each of the 156,571 watersheds, ML-estimated *D*_*d*_ is compared with *D*_*d*_ of the dense river network generated with the 1 km^2^ threshold. If the latter is greater (meaning the river network is too dense compared to what is expected), the river network is trimmed by continuously eliminating stream segments with the smallest drainage areas, until the watershed’s *D*_*d*_ becomes close enough to ML-estimated *D*_*d*_. Otherwise, the dense river network is not trimmed assuming 1 km^2^ is the finest threshold we can achieve with this new global hydrography, which is reasonable given the DEM resolution as well as the computational constraints (*Section 2.3*).

## Data Records

We summarize the generated data records^34^ in Table 1, in which the data category, attributes, type, number of objects, and file sizes are presented. The data downloading is facilitated through the 61 level-02 basins; their geographic locations are provided in Fig. S9.

## Technical Validation

### Centerline accuracy assessment

**Fig. 2:**
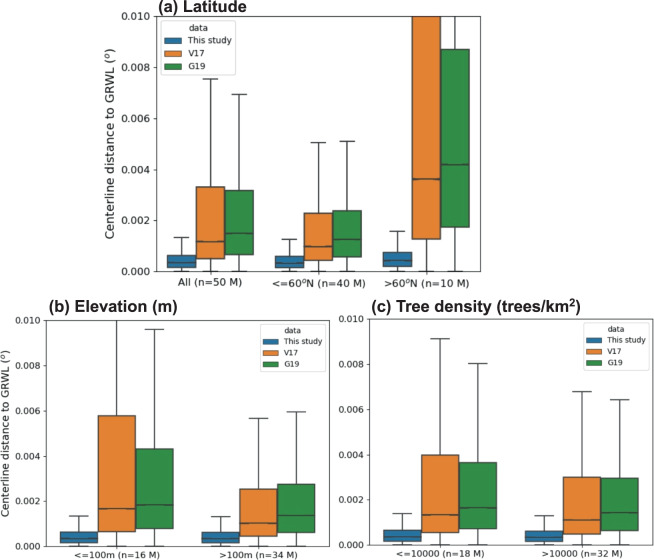
Assessment of the centerline accuracy compared against the Landsat-derived river centerline at ~50 million data sample locations. The boxplot summarizes the distance between the Landsat centerline points to the nearest MERIT flowlines (blue), the V17 flowlines (orange), and the G19 flowlines (green) in the unit of decimal degrees; n shows the number of GRWL centerline points involved in the calculation; M = million. (**a**) separates rivers below/above 60°N, because 60°N is a critical latitude above which SRTM DEM was previously lacking. (**b**) separates rivers below/above 100 m, as below 100 m is considered flat regions where challenges for DEM-extracted flowlines exist. (**c**) separates rivers according to tree density, as regions with high tree density are expected to have larger biases in DEMs. Global tree density data is from Crowther *et al*.^40^.

We first assess the centerline accuracy of the new hydrography dataset by comparing it against GRWL^20^, the Landsat-derived centerlines at over 50 million cross sections globally. Headwater streams narrower than 30 m are not explicitly included in this analysis due to a lack of good reference data for small rivers, which remains an important future task^33^. It must be noted that although GRWL only covers rivers wider than 30 m, the unprecedented number of cross sections (>50 M) and its global coverage makes GRWL the best available reference data to use (i.e., the analysis is not biased towards specific regions). In addition, since the creation of GRWL is independent of DEM-based methods, it can provide us with an objective comparison. To benchmark the assessment, we additionally incorporate the HydroSHEDS 3-arcsec and 15-arcsec data developed by Verdin *et al*.^35^ and Grill *et al*.^14^, respectively (hereafter referred as V17 and G19), for a comparison. More specifically, for each of the ~50 million centerline locations in GRWL, the closest MERIT river reach is found by searching a radius of 10 km; the same practice is done for the closest V17 and G19 reaches. Then, the closest distances between GRWL and the DEM-based flowlines (in decimal degrees) are summarized as centerline errors (measured by “Centerline distance to GRWL”) (Fig. 2), where detailed error analyses separating different latitudes (Fig. 2a), elevation bands (Fig. 2b), and tree density (Fig. 2c) are also performed.

Figure 2a clearly shows that our river network consistently has the smallest centerline error across different latitudinal bands. For the Arctic rivers above 60°N where the SRTM DEM is limited in offering accurate flowline depictions, the MERIT-Hydro derived vector river flowlines (this study) provide the most pronounced gains. More reduced centerline errors can also be observed for flat regions (i.e., elevation ≤100 m) than higher-altitude regions (Fig. 2b, elevation >100 m) compared to V17 and G19. Additionally, since tree canopies are also a source of bias for DEMs^16^, we further separate the assessment with tree density. In Fig. 2c, gains from using MERIT Hydro are seen, but for regions with high tree density (>10,000 trees/km^2^), the gains seem to be similar to those from low tree density regions. Overall, it is promising to see much improved centerline accuracy in our dataset across different latitudes, elevations, and tree densities. The median improvement of 0.001° to 0.004° corresponds to up to approximately 400 meters depending on the latitude, and this is a significant distance that can play a big role in the global hydrodynamic modeling and flood inundation mapping accuracy, both of which require accurate depictions of river centerline locations.

### Spatial variability of drainage density assessment

**Fig. 3:**
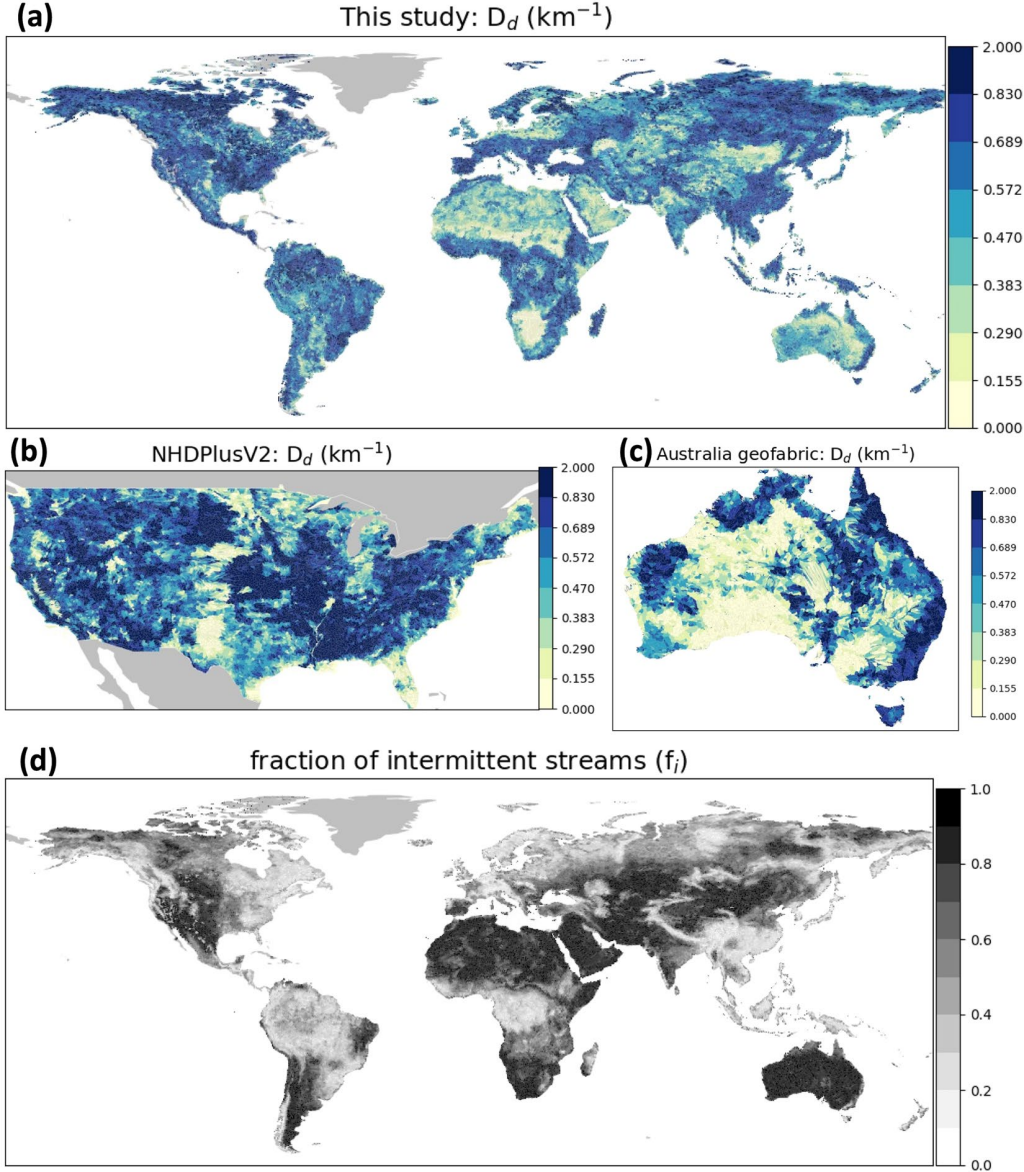
Assessment of the *D*_*d*_ spatial pattern. (**a**) shows the ML-estimated *D*_*d*_ derived in this study. (**b**,**c**) show the reference *D*_*d*_ for CONUS and Australia, as summarized from the NHDPlusV2 data and the Australian geofabrics (accounting for both perennial and intermittent streams), respectively. (**d**) shows the fraction of intermittent streams (*f*_*i*_, defined as length of intermittent streams divided by total lengths of the stream network) globally, as derived in this study. *D*_*d*_ spatial patterns of two other hydrography datasets based on HydroSHEDS (V17 and G19) are shown in Fig. S8.

We also assess the ML-estimated *D*_*d*_ by comparing it with selected high-quality regional hydrography datasets, including the NHDPlusV2^20^ (also used in training the ML model), the Australia geofabric^22^, as well as several field-mapped river network data^12,36^ (Fig. 3). These are used as the reference because they are well documented and validated previously.

Spatially, the estimated *D*_*d*_ seems to reasonably reflect the dominant climatic controls, where wetter regions generally show greater *D*_*d*_ above 0.6 km^−1^, such as the eastern US, southeastern China, the Amazon river basin, the Congo river basin, and part of the arctic basins (Fig. 3a). This is contrasted with drier regions such as the central US, central Asia, middle east, northern and southwestern Africa, Australia, the Tibet, and the Mongolia, where *D*_*d*_ is generally less than 0.3 km^−1^. In the US, relatively lower *D*_*d*_ in some local parts of Florida, the Great Plains, and California shown in the reference data (Fig. 3b) are reasonably captured, albeit with slight positive biases (comparing Fig. 3a with Fig. S3c). In Australia, the higher *D*_*d*_ along the northern and eastern coast is also well reflected (comparing Fig. 3a with Fig. 3c). Although ML seems not perfectly capturing small-scale *D*_*d*_ in some locations, we note that the overall improvement is significant compared with V17, which uses a 250 km^2^ threshold based on the 3 s HydroSHEDS data^35^ and thus delineating much less channels than reality. It also compares much more favorably with G19, which uses a 0.1 m^3^/s or 10 km^2^ threshold based on the 15 s HydroSHEDS data^14^ (presented in Fig. S8). In order to better inform users on areas with potentially greater *D*_*d*_ uncertainties due to the difficulty in determining intermittent streams in our reference data NHDPlusV2 (see Text S2 for caveat in addressing intermittent streams), we also present the ML-estimated patterns of the fraction of intermittent streams (*f*_*i*_) in Fig. 3d (see reference *f*_*i*_ in Fig. S3d). It can be seen that in the newly delineated global hydrography, over 80% of the total drainage lengths are intermittent streams in the western US, northern and south Africa, inland Australia, middle east, and some central Asia areas. These regions have very low perennial *D*_*d*_ due to a lack of constant precipitation inputs (e.g., the western US, Fig. S3c), but their geomorphic *D*_*d*_ is relatively high because both intermittent and perennial channels are accounted here. However, we must note that *f*_*i*_ is highly uncertain and our estimates have not been validated due to a lack of reference data. While our study provides a possible estimate of *f*_*i*_ globally, *f*_*i*_ in our training data is also subject to uncertainties. Therefore, future work remains to be done to better resolve this problem.

**Fig. 4:**
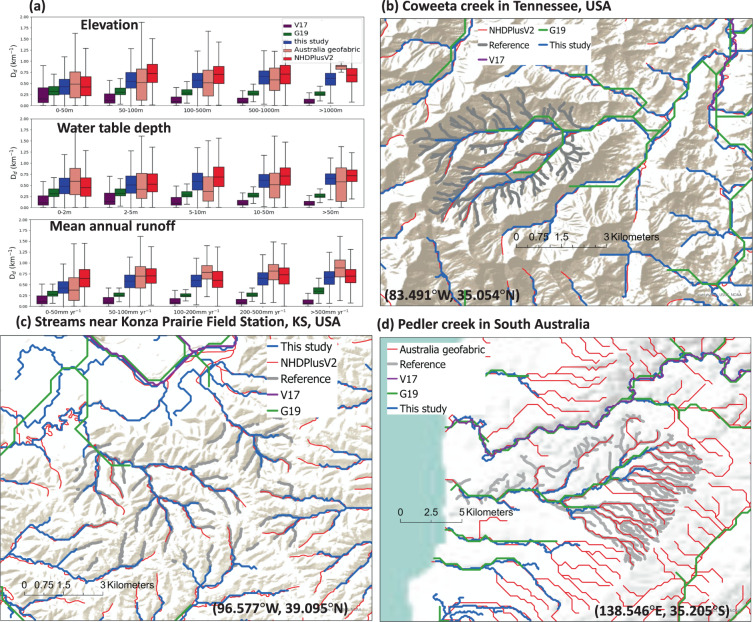
Further assessment of the *D*_*d*_ patterns. (**a**) shows *D*_*d*_ as a function of mean elevation, water table depth, and mean annual runoff in different datasets. (**b**–**d**) shows examples where the newly delineated global river network data are compared against field-informed river network data (i.e., “Reference” in grey). (**b**) is the Coweeta Creek in Tennessee; the reference field-based river network is obtained from Benstead and Leigh;^12^ (**c**) shows the Konza Prairie Field Station in Kansas; the reference is obtained from http://lter.konza.ksu.edu/data/gis; and (**d**) shows the Pedler creek in South Australia; the reference is obtained by the field work from Shanafield *et al*.^36^ V17 is derived from 3 s HydroSHEDS data with a channelization threshold of 250 km^2^. G19 is derived from 15 s HydroSHEDS data with a channelization threshold of 0.1m^3^/s or 10 km^2^. The dataset of this study is derived from 3 s MERIT data with a channelization threshold defined by machine learning estimates, topped at 1 km^2^.

In general, referencing against two continental-scale hydrography datasets NHDPlusV2 and the Australia geofabric, our new global hydrography shows consistently better *D*_*d*_ as a function of elevation, water table depth (WTD), and mean annual runoff, compared to both V17 and G19 (Fig. 4a). V17 significantly underestimates *D*_*d*_ due to its 250 km^2^ channelization threshold. G19 slightly alleviates this problem with a finer threshold (0.1 m^3^/s or 10 km^2^), but it does not reflect the *D*_*d*_ variability across different topographic, WTD, and runoff conditions. By using variable channelization thresholds defined by ML estimates here, the new hydrography can address the problem better. In addition, our dataset also demonstrates much improved capability in capturing headwaters as compared against several small-scale field-informed reference river network datasets collected in the US and Australia (grey thick lines in Fig. 4b–d). Although under-representations of headwater streams are still found, it is expected due to the use of the channelization threshold topped at 1km^2^ while we note it already outperforms state-of-the-art global hydrography datasets. Therefore, we expect this new global hydrography to be used to facilitate refined quantifications of global CO_2_ emissions from rivers^37^, geomorphological and ecohydrological analyses^38^, and global hydrodynamic modeling^3^, where more realistic density of hillslopes and river channels (Fig. 3a) and improved channel travel time representations may offer new scientific insights. Moreover, river longitudinal concave profile analysis^39^ may also benefit from the enhanced-accuracy river centerlines of this study (Fig. 2). The new global hydrography dataset (MERIT Hydro–Vector) is publicly shared at *figshare* (10.6084/m9.figshare.c.5052635) and can be downloaded separately for 61 level-02 basins shown in Fig. S9. In accordance with the MERIT Hydro data, the MERIT-Hydro–Vector data version is v1.0.1a (v.1.0.1 represents the version for MERIT Hydro and the letter represents the version for the vector product).

## Supplementary information

Supplementary Information

## Data Availability

The new global vector-based hydrography dataset, consisting of basins, watersheds, and river networks of variable and constant *D*_*d*_, is produced using Python v3.7.3 and the TauDEM software v5.3.8. All computations are completed using the Della high-performance computing clusters at Princeton University. For geospatial analysis, we use the freely available GeoPandas library in Python; for some figure displaying purposes, we use the ArcPro version 2.4.1. Key Python scripts developed for this work are openly shared with the scientific community at Github: https://github.com/peironglinlin/Variable_drainage_density.
