# Supplementary material for: A new vector-based global river network dataset accounting for variable drainage density
Source: Sci Data. 2021 Jan 26;8:28. doi: 10.1038/s41597-021-00819-9 (PMC7838288; doi:10.1038/s41597-021-00819-9)
Supplement: Supplementary file 1 — Supplementary Information [file 41597_2021_819_MOESM1_ESM.pdf]

Supporting Information for

Lin et al.

<u>Content</u>

Texts S1 – S2

Figures S1 – S9

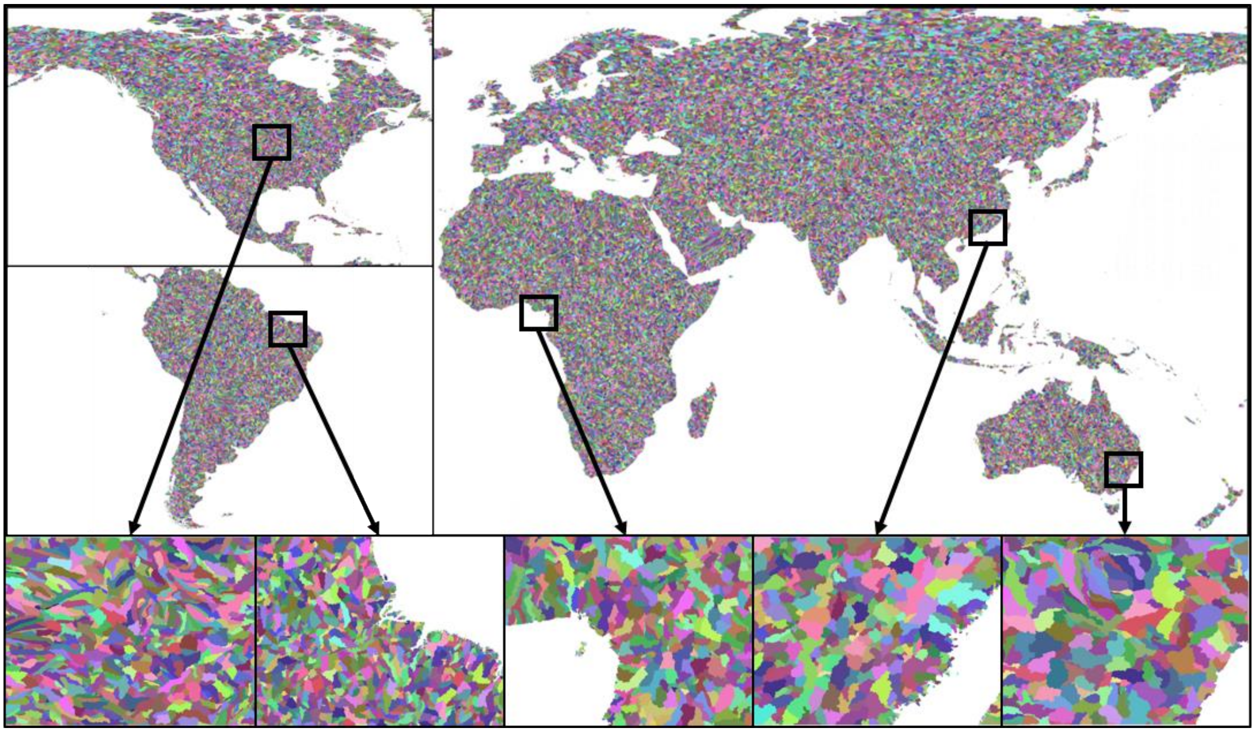

Figure S1. The global watersheds (156,571) delineated by this study. Colors are randomly assigned based on the unique watershed ID. The median watershed size is 461 km^2, which is equivalent to HydroBASINS level-08 basin size (median: 475.7 km^2).

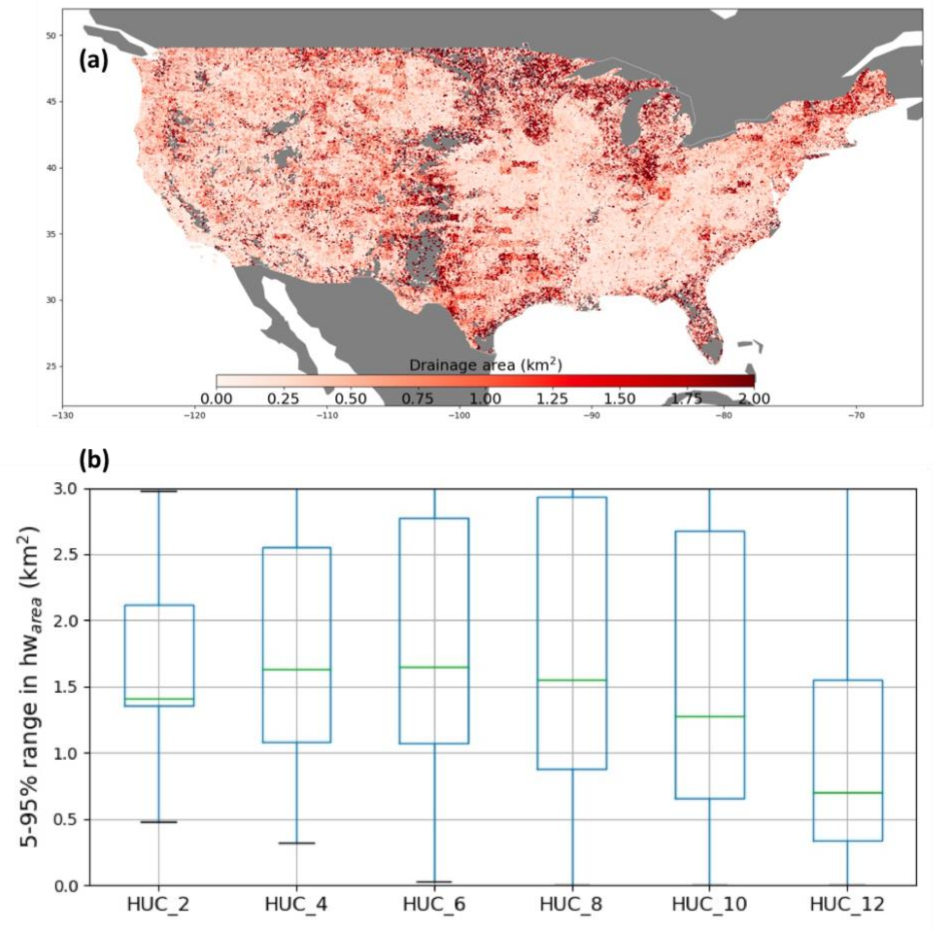

Figure S2. Spatial variability of the drainage areas for all channel heads (hw_{area}, km^2) in NHDPlusV2. (a) Spatial plot of hw_{area}. Sample size n = 891,570, where only "natural streams" are retained and reaches labeled "Pipelines", "Canal Ditches", "Coastlines", and "Aritificial Paths" are all removed. (b) Boxplot of hw_{area} range (5% – 95%) as a function of the Hydrologic Unit Codes (HUC) regions: the size ranges from HUC_2 (median: 285,025.45 km^2) to HUC_12. (median: 88.23 km^2). The boxplot summarizes the minimum, lower quartile, median, upper quartile, and maximum hw_{area} range in each HUC level.

Text S1. Spatial variability of the channel head drainage area in NHDPlusV2. Although one can obtain channel head drainage areas (hw_{area}, km^2) from NHDPlusV2, which is straightforward to use in determining the channelization threshold, we do not directly use it here because of the following reasons (watershed-level D_d is used instead; see *Section 2.5* for more details). First, by observing the spatial pattern of hw_{area} across the US (**Fig. S2a**), we found that the drainage areas are sometimes not varying smoothly, and patterns of county boundaries can be found. This suggests the compilation of NHDPlusV2 channel heads is likely influenced by different collection standards and data sources adopted across different counties in the US[21]. In fact, subjective decisions on channel head definitions are almost always inevitable in any geofabrics dataset[26], unless field surveys are consistently conducted[11] over a large spatial domain, yet this is unlikely to achieve. Second, many of the channel head drainage areas are under 1 km^2 (the smallest being ~900 m^2). However, given the constraints by DEM resolution (~90 m), the finest channelization threshold we can achieve is well beyond 8100 m^2 (~90 m × 90 m) – the smaller the threshold is, the more uncertainties in the delineated channel lines. Therefore, we were informed by these earlier assessments that in using NHDPlusV2 or any other high-quality regional networks as our reference data for estimating D_d, we do not aim, nor is it possible, to achieve the headwaters as defined by these geofabrics or those surveyed in the field work. Instead, we only attempt to achieve the watershed-level D_d variability globally, under the reasonable assumption that the watershed-by-watershed spatial variability of D_d is well represented in these regional geofabrics such as the NHDPlusV2[31].

To determine what is the appropriate watershed size to use, we conduct an additional assessment of the hw_{area} range as a function of the watershed size, defined by the HUC units (**Fig. S2b**). Ideally, the most representative size should have the smallest hw_{area} range (meaning that hw_{area} does not vary much within the same HUC unit). However, smaller watershed size also means globally we need to split the global basins into smaller units to apply variable D_d, which can significantly increase the computational time. To leverage the computation and the representativeness of the watershed size, we eventually choose HUC_10 (median: 470.21 km^2) to train the spatial variability of D_d, and ensuing geospatial analyses over the continental United States (CONUS) are all conducted at the HUC_10 level.

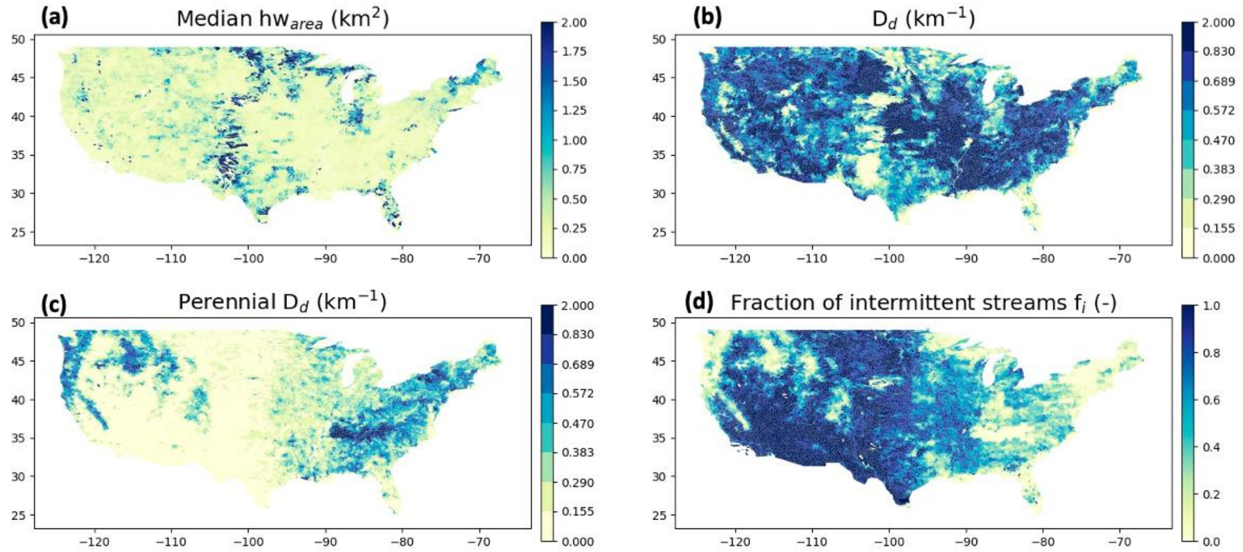

Figure S3. Spatial patterns of the (a) median hw_{area}, (b) D_d, which accounts for both perennial [code: 46006] and intermittent streams [code: 46003] in NHDPlusV2, (c) perennial D_d, and (d) fraction of intermittent streams (f_i, length of intermittent streams divided by the total length of river networks) at the HUC-10 level.

Text S2. Covariates selected to estimate D_d spatial variability. The selection of the covariates, or predictors of D_d, is based on our physical knowledge on what controls D_d variability. They include (1) climatic factors such as aridity index (*AI*), (2) topographic factors such as mean and standard deviation of elevation (*topo* & $topo_{std}$), (3) hydrologic factors such as mean runoff (Q_{mean}), water table depth (*WTD*), soil clay/silt/sand mass percentage content (*CLY, SLT, SND*), leaf area index (*LAI*), and urban fraction (*Urb*), and (4) geologic factors such as bedrock hydraulic conductivity (*K*) and porosity (*P*). The calculation of these covariates follow Lin et al.[33] where details on data sources are provided, except here the zones for statistical calculations are the HUC-10 units for training and the watersheds in **Fig. S1** for extrapolation. Compared to the past studies that only used runoff[2,14] or topographic factors[5,9] for channelization research, here we explicitly incorporate more potential factors in a data-driven approach to see if they can offer new insights into what controls D_d variability and thus improving the prediction skill of D_d.

As can be seen from **Figs. S4 & S5**, these factors show some spatial correspondences with D_d, however, highly nonlinear relationships are exhibited in the scatter plots. There are some expected tendencies for higher D_d to appear with higher Q_{mean} and silt content, and lower D_d to appear with higher sand content, however, the overall relationships between D_d and other factors seem very complex. The main reason is that what we try to extrapolate is the D_d that accounts for both perennial and intermittent streams (**Fig. S3b**), yet occurrence of intermittent streams is a result of more complex physical mechanisms and its determination in any geofabric data potentially involves more subjectivity[40]. By contrast, we see that the relationships between covariates and perennial D_d (**Fig. S3c**) have much more intuitive and linear patterns (**Fig. S6**), suggesting that the occurrence of perennial streams is much more predictable than that of intermittent streams. Despite the difficulty, we still aim to extrapolate D_d (instead of perennial D_d) patterns in this study because of our goal to delineate all potential channels, including intermittent streams, in the new global hydrography. To give users more information, we also extrapolate fraction of intermittent streams (f_i) for each watershed globally, which can inform users about places with higher f_i that are also potential areas with higher uncertainty.

Feature importance plot in **Fig. S7** suggests that in addition to topography, Q_{mean}, and climatic aridity index, water table depth and soil silt/clay/sand content are also important predictors of D_d. This suggests that conventional approaches (only considering topography, runoff, or climate aridity) may need to be revised to better study the channelization criteria.

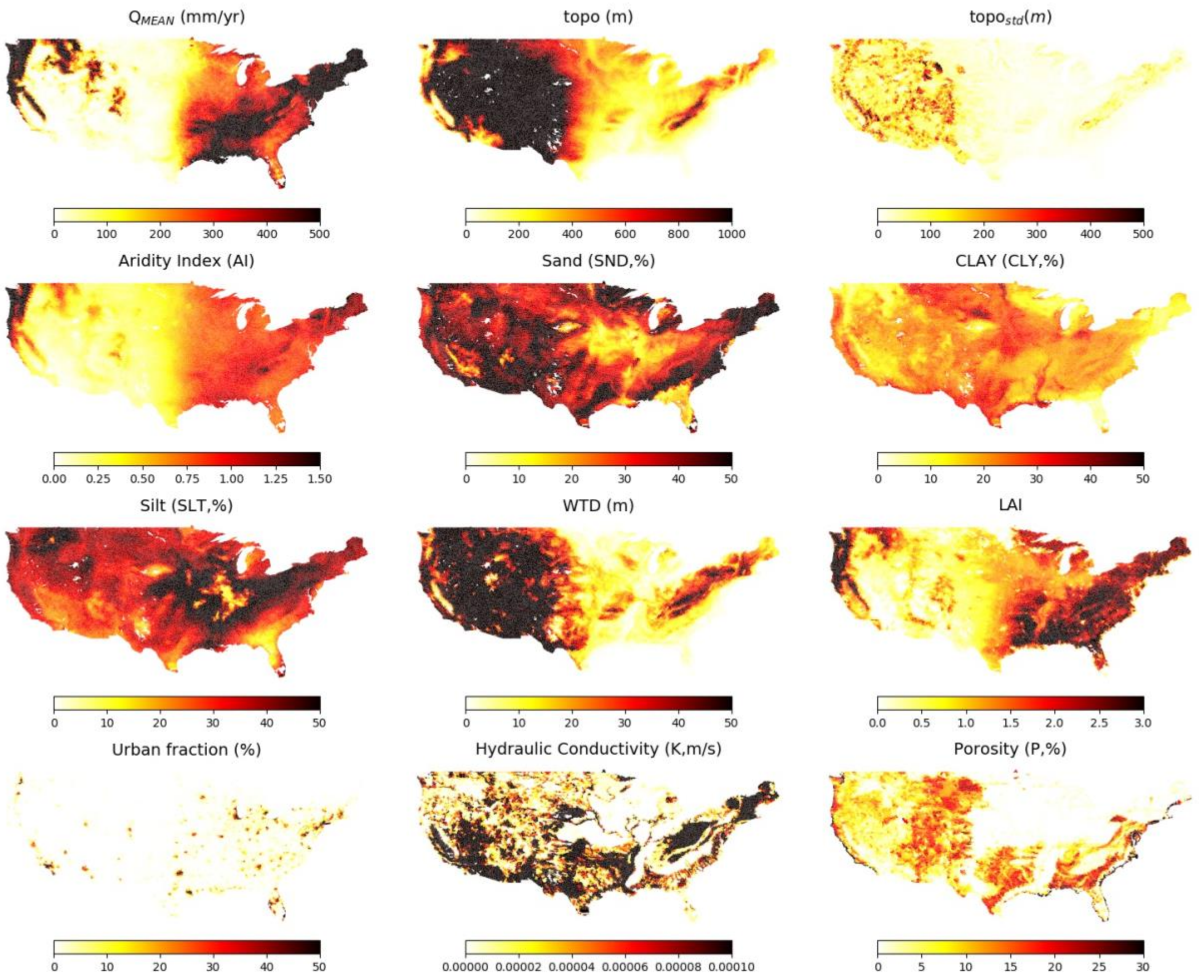

Figure S4. Environmental covariates used to estimate drainage density.

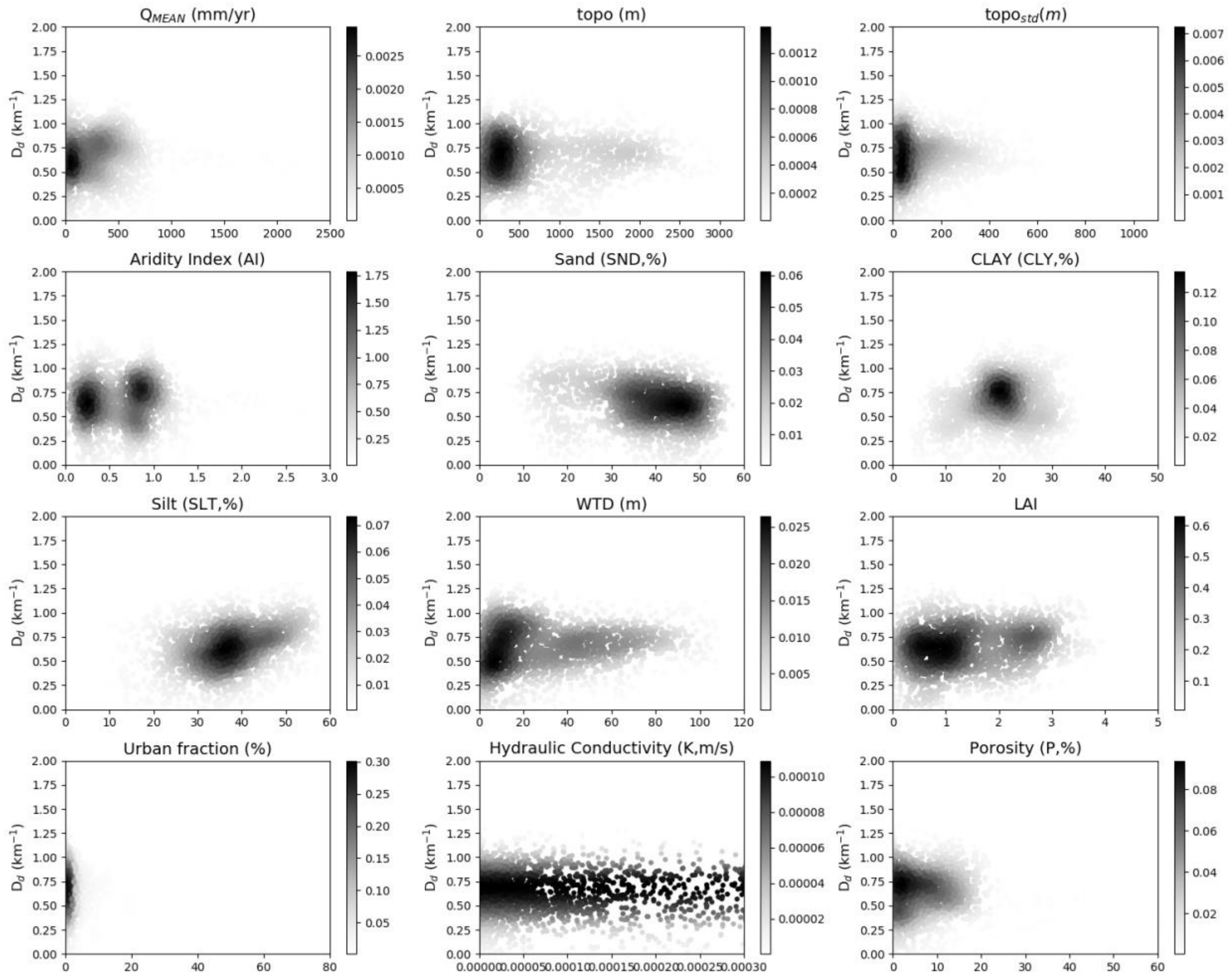

Figure S5. Density scatter plot between environmental covariates and D_d.

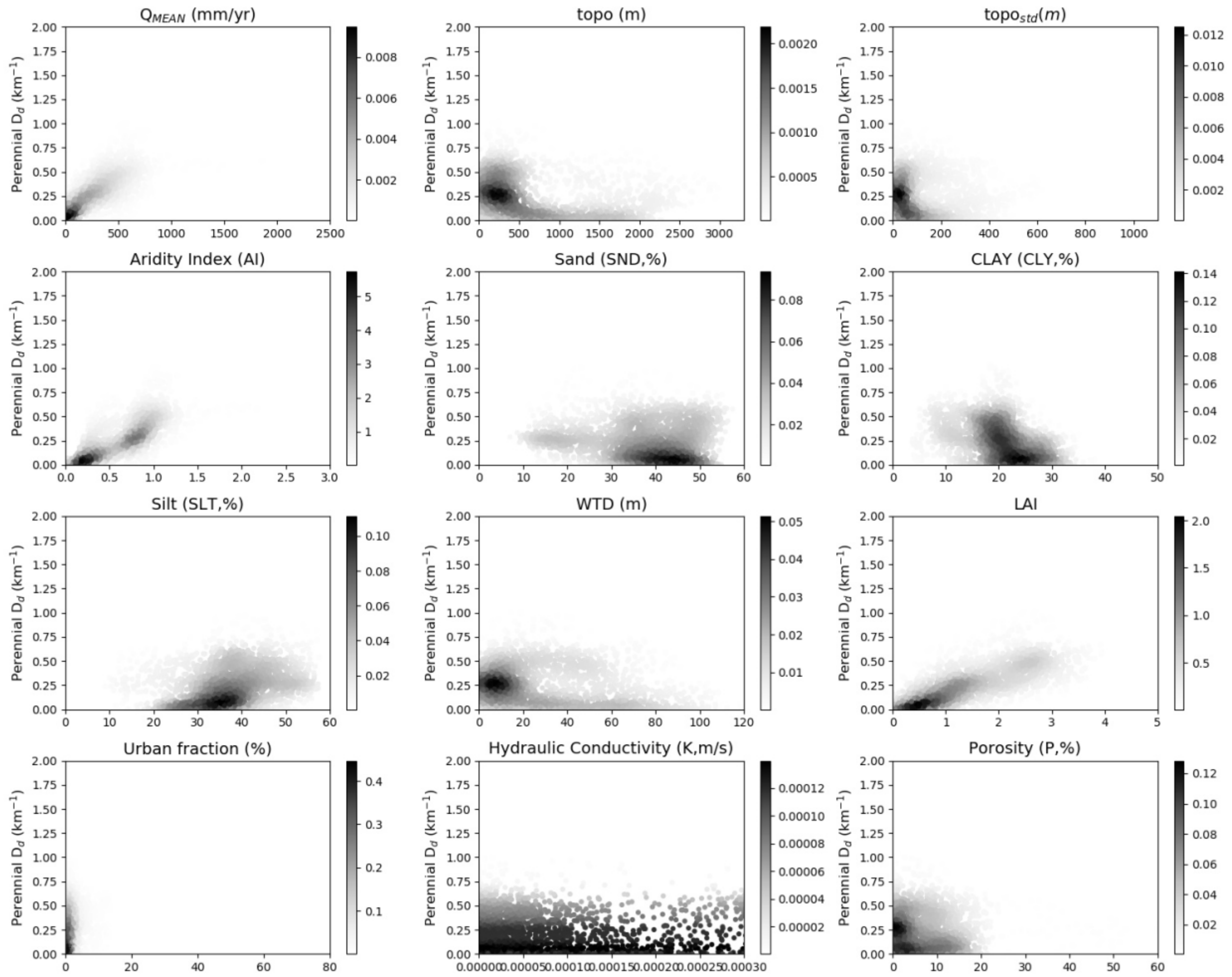

Figure S6. Density scatter plot between environmental covariates and perennial D_d.

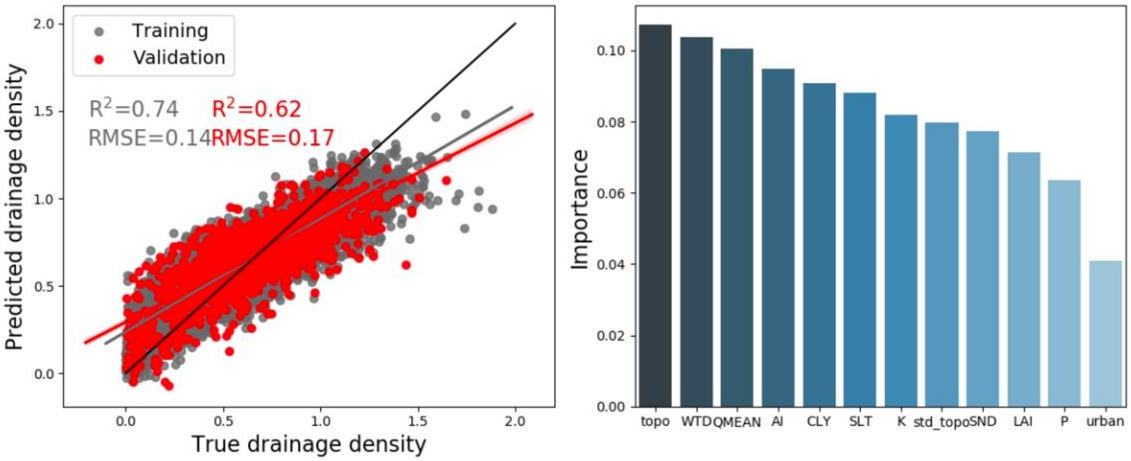

Figure S7. Training and validation statistics of the machine learning method in estimating D_d (left). The feature importance is shown on the right.

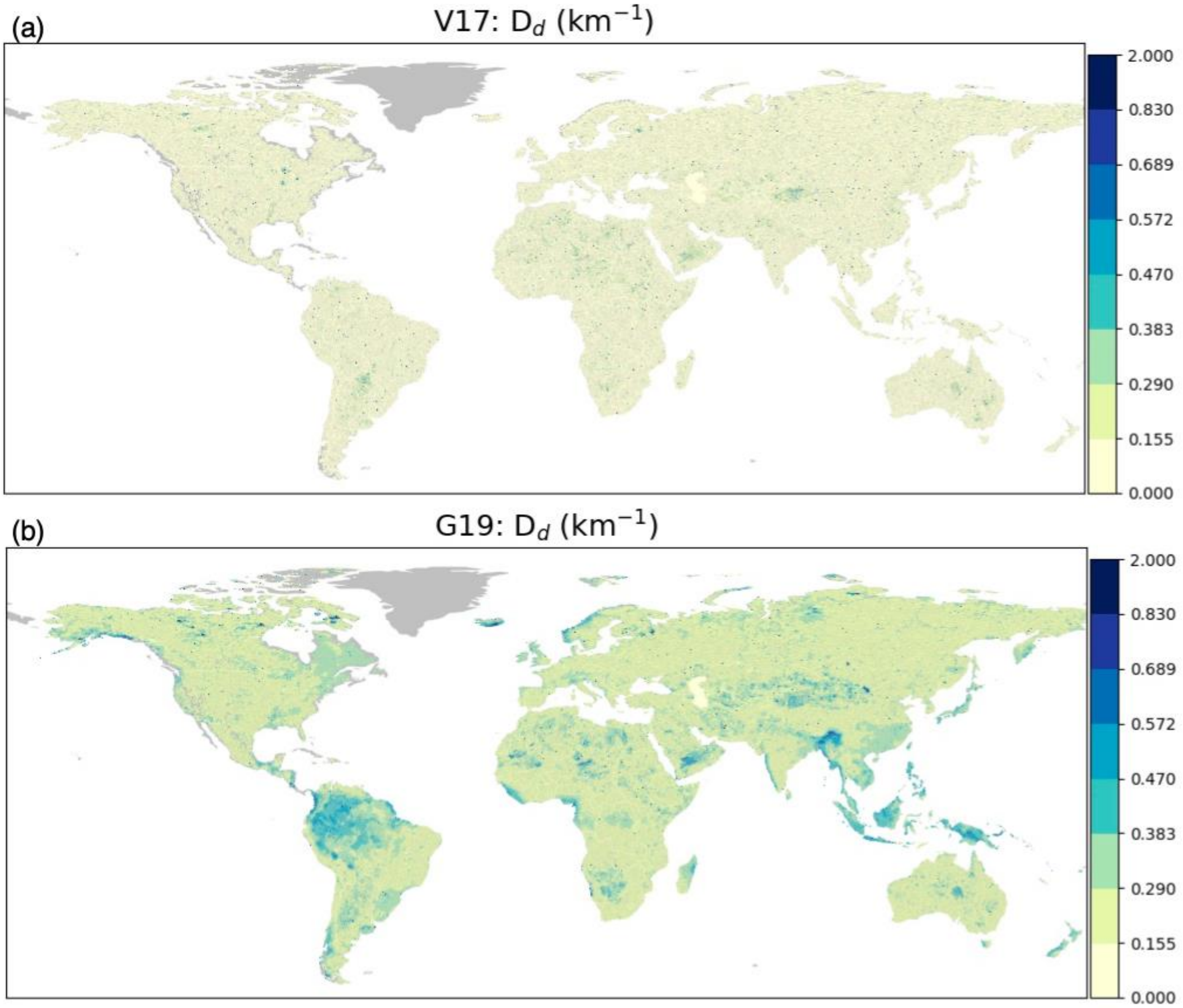

Figure S8. Drainage density (D_d, km^{-1}) of the hydrography datasets by (a) Verdin (2017), referred as V17, and (b) Grill et al. (2019), referred as G19. Note that V17 used 3s HydroSHEDS data to delineate the vector river network with a channelization threshold of 250 km^2. G19 used 15s HydroSHEDS data to delineate the vector river network with a channelization threshold of 0.1 m^3/s or 10 km^2.

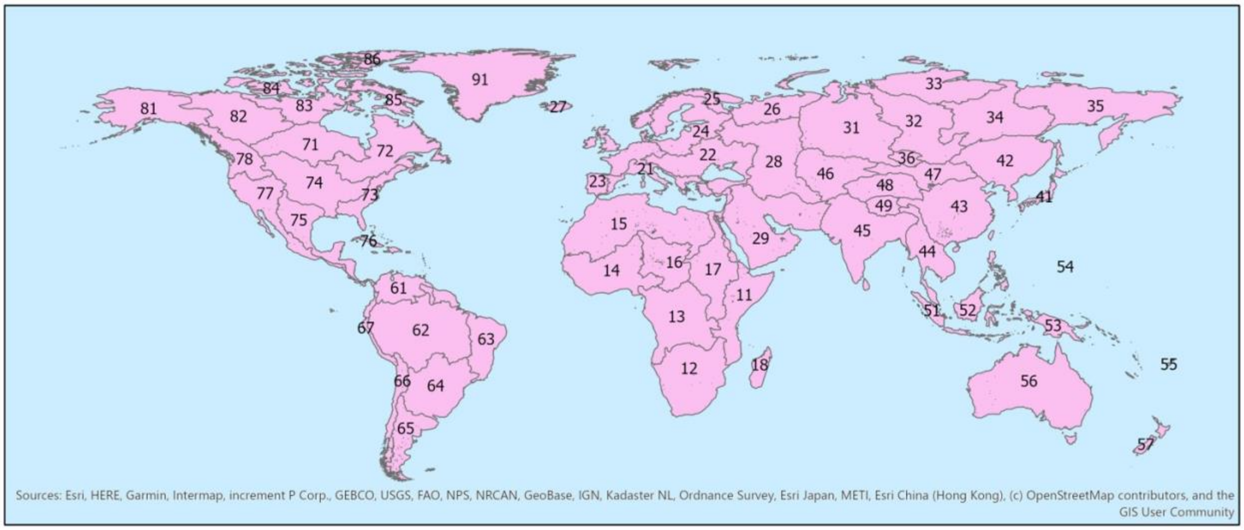

Figure S9. Level-02 basin numbers for the river network data organization and downloading.
